# Low-Power Wireless Sensor Module for Machine Learning-Based Continuous Monitoring of Nuclear Power Plants

**DOI:** 10.3390/s24134209

**Published:** 2024-06-28

**Authors:** Jae-Cheol Lee, You-Rak Choi, Doyeob Yeo, Sangook Moon

**Affiliations:** 1 Nuclear System Integrity Sensing and Diagnosis Division, Korea Atomic Energy Research Institute (KAERI), 989-111 Daedeok-daero, Yuseong, Daejeon 34057, Republic of Korea; jclee2@kaeri.re.kr (J.-C.L.); yrchoi@kaeri.re.kr (Y.-R.C.); yeody@kaeri.re.kr (D.Y.); 2 Department of Electrical and Electronic Engineering, Mokwon University, 88 Doanbuk-ro, Seo-gu, Daejeon 35349, Republic of Korea

**Keywords:** low-power wireless monitoring, nuclear power plants, heterodyne frequency conversion, edge computing, predictive maintenance

## Abstract

This paper introduces the novel design and implementation of a low-power wireless monitoring system designed for nuclear power plants, aiming to enhance safety and operational efficiency. By utilizing advanced signal-processing techniques and energy-efficient technologies, the system supports real-time, continuous monitoring without the need for frequent battery replacements. This addresses the high costs and risks associated with traditional wired monitoring methods. The system focuses on acoustic and ultrasonic analysis, capturing sound using microphones and processing these signals through heterodyne frequency conversion for effective signal management, accommodating low-power consumption through down-conversion. Integrated with edge computing, the system processes data locally at the sensor level, optimizing response times to anomalies and reducing network load. Practical implementation shows significant reductions in maintenance overheads and environmental impact, thereby enhancing the reliability and safety of nuclear power plant operations. The study also sets the groundwork for future integration of sophisticated machine learning algorithms to advance predictive maintenance capabilities in nuclear energy management.

## 1. Introduction

Nuclear power plants (NPPs) utilize the fission of atomic nuclei to generate heat, which is subsequently converted into electrical energy. This process is critically facilitated by two distinct yet interdependent systems: the primary and secondary coolant systems.

The primary system is designed to manage the heat generated from nuclear fission within the reactor. It involves a closed loop where a coolant (typically water) is heated by the reactor’s core. The primary system manages the reactor-generated heat, circulating the coolant that absorbs heat without vaporizing, transferring it via a steam generator to the secondary system. Here, the heat converts water into high-pressure steam through long pipes with a maximum possible surface area, which then drives turbines to produce electricity. After powering the turbines, the steam condenses back to water, which is recirculated to the steam generator, completing the cycle [1,2]. While these systems operate independently, their efficiency and safety are interlinked, with any degradation or failure in the pipe systems posing severe risks—from radioactive leaks that threaten public health and the environment [3,4], to steam generator malfunctions that could trigger explosions [5]. Additionally, any significant damage might necessitate a shutdown of the plant operations, disrupting electricity supply and causing widespread economic impacts. To clearly articulate the objectives of this paper from the outset, we focus on developing a novel low-power wireless monitoring system for isolated pipe systems, with a specific emphasis on nuclear power plants. This research is designed to enhance safety protocols and reduce the economic impacts of equipment failures by enabling real-time, continuous monitoring without the need for frequent battery replacements.

**Figure 1:**
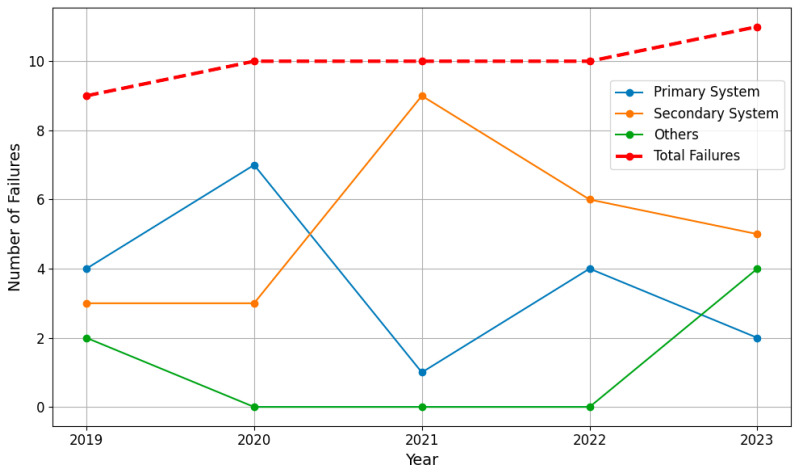
NPP fault sources and total number of faults (red dashed line) from 2019 to 2023 in South Korea [6].

Figure 1 illustrates that the steady increase in equipment failures in Korean nuclear power plants between 2019 and 2023 requires the urgent need for enhanced diagnostic methodologies as these facilities continue to age [6]. However, the inherent risks associated with radioactive exposure complicate the deployment of personnel for on-site inspections and maintenance, emphasizing a critical need for alternative monitoring approaches.

While wired sensor networks have traditionally been utilized for structural monitoring, their deployment and maintenance introduce significant challenges and costs, particularly related to the extensive wiring required which contributes majorly to the operational expenditures of NPPs [7]. The integration of wireless sensor technologies presents a viable solution to these issues, particularly through the reduction of installation complexity and associated costs. Prominent applicable wireless technologies include Bluetooth, Zigbee, Wi-Fi, LoRa, WirelessHART, Terahertz, infrared (IR) communication and more [8,9]. However, the adoption of wireless technologies involves using batteries as their power source, necessitating periodic replacement or recharging. This requirement introduces additional maintenance tasks that must be managed over the long term, presenting significant challenges in the administration of large-scale systems [10]. Among the methods to mitigate these challenges such as energy harvesting technology, advanced battery technology, energy-efficient communication technology, etc. [11,12], we focus on development and implementation of low-power wireless monitoring systems, not only for reducing maintenance costs but also for ensuring continuous and reliable monitoring to preemptively address potential failures and minimize downtime. The technology will be described in Section 4 and Section 5.

This paper proposes a novel low-power wireless monitoring system designed specifically for NPP applications. The system leverages advanced signal-processing techniques and energy-efficient technologies to enable long-term, real-time monitoring without frequent battery replacements, thereby enhancing safety protocols and eventually reducing the economic impact of plant shutdowns due to equipment failures. As we delve into the technical aspects of our proposed system, it is important to contextualize the design choices made. These decisions are driven by the critical challenges identified earlier, such as the need for reducing maintenance overheads and improving the reliability of monitoring in the hostile environments typical of nuclear power plants. By integrating state-of-the-art signal-processing techniques and energy-efficient technologies, our design aims to address these challenges effectively.

## 2. Heterodyne Frequency Conversion

Heterodyne frequency conversion is a fundamental signal-processing technique used primarily in communication systems to shift the frequency of an electromagnetic signal [13]. This method is pivotal in various applications such as radio, television broadcasting and satellite communications, and the idea starts with Equation (1).

(1) $$\cos\omega_{1}t\cdot \cos\omega_{2}t=\frac{1}{2}(\cos\left(\omega_{1}-\omega_{2}\right)t+\cos\left(\omega_{1}+\omega_{2}\right)t),$$

where $\omega_{1}$ and $\omega_{2}$ are the frequencies of input signals, and *t* is time.

**Figure 2:**
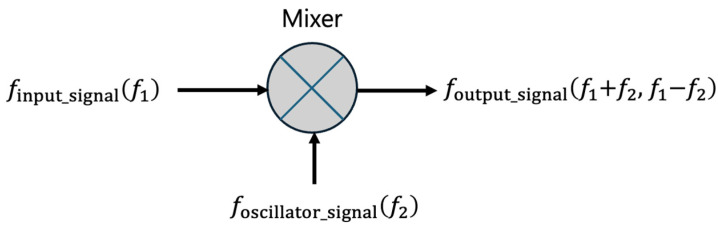
Heterodyne frequency conversion.

The process begins with two signal functions: the input signal and a locally generated signal from a local oscillator (LO). The input signal function, which carries the desired information, has a frequency *f*_1_ that is usually not suitable for direct processing either because it is too high or not aligned with the system’s capabilities. The local oscillator generates a second signal function at a predetermined frequency *f*_2_. The core operation in heterodyne frequency conversion involves the multiplication of the input signal by the local oscillator signal. This multiplication occurs in a device called a mixer. We will explain the mixer later in this paper. The mixer outputs a signal that contains components at both the sum (*f*_1_ + *f*_2_) and difference (*f*_1_ − *f*_2_) of the frequencies of the two input signals, where *f*_1_ is the frequency of the input signal, and *f*_2_ is the frequency of the local oscillator signal as shown in Figure 2. 

This can be expressed in mathematical equations as follows:

(2) $$x\left(t\right)\cdot y\left(t\right)=A\cos\left(2\pi f_{1}t\right)\cdot B\cos\left(2\pi f_{2}t\right)=\frac{AB}{2}\left[\cos\left(2\pi (f_{1}+f_{2}\right)t+\cos\left(2\pi (f_{1}-f_{2}\right)t)\right],$$

where $x\left(t\right)=A\cos\left(2\pi f_{1}t\right)$ is the input signal and $y\left(t\right)=B\cos\left(2\pi f_{2}t\right)$ is the local oscillator signal.

The next step in the heterodyne process involves filtering out one of these frequencies, typically either of the sum or the difference, depending on the desired outcome. The choice of frequency depends on whether an up-conversion or down-conversion is needed. Up-conversion increases the frequency for purposes like transmission over certain media, while down-conversion reduces the frequency for easier processing and demodulation. Applying this technique enables the measurement of anomalous signals at significantly lower frequency bands compared to the original frequency range. Consequently, the associated filters, amplifiers (AMP) and A/D converters employed alongside this method can operate at these reduced frequencies, thereby substantially decreasing power consumption. This efficiency is critical in enhancing the sustainability and cost-effectiveness of our target system. 

## 3. Wireless Monitoring Method

### 3.1. Edge Sensor Operation in Wireless Monitoring

**Figure 3:**
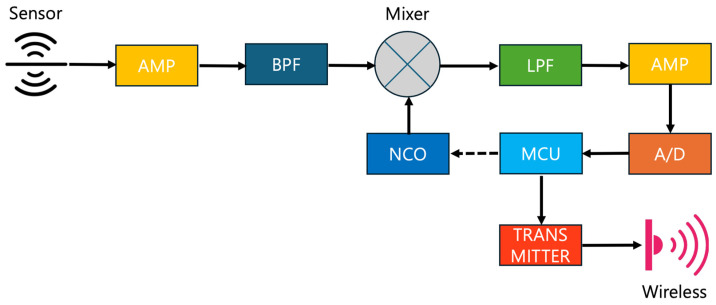
Proposed edge sensor operational diagram.

Figure 3 illustrates the essential operations and connections of the low-power wireless monitoring device proposed in this paper. The system comprises sensors, a first amplifier, a bandpass filter, a numerically controlled oscillator (NCO), a mixer, a lowpass filter, a second amplifier, an analog-to-digital converter (ADC), a microcontroller unit (MCU), memory and a transmitter. Signals collected by sensors are first enhanced and improved in quality by the first amplifier, then passed through the bandpass filter to eliminate noise and emphasize the desired signals. These signals, initially collected in the high-frequency band, are inputted into the mixer, along with a frequency generated by the real-time programmable NCO for acquiring low-frequency mixer output. The output from the mixer, a nonlinear component, contains both low and high-frequency signals. Since our interest lies in extracting the low-frequency band signals, these are isolated by passing through the lowpass filter, which removes the high-frequency content. The selected low-frequency signals are then further enhanced and improved in quality by the second amplifier. These signals are subsequently converted into digital data by the ADC to enable processing by the MCU. The converted digital data are analyzed by the MCU to distinguish between normal and anomalous signals. In case of anomalies, the signals are then transmitted wirelessly to communicate the corresponding information to the edge server for in-depth examination. Otherwise, to save power, no full communication is established; instead, a *sensor_alive* packet containing only the header is sent every 30 min. Since abnormal signals have been most effectively detected at 38 kHz based on empirical data obtained from years of monitoring reactors, the determination of suspected anomalies is based on the average value of spectral data summed between 30 kHz and 45 kHz. The threshold is set based on the average value mixed with background noise under experimental conditions. For example, a signal is considered anomalous if it deviates by 20% or 30% from the background noise average. In this way, it reduces additional handling needs, thus decreasing network traffic and computational load. 

### 3.2. Edge Sensor, Edge Server and Cloud

If a signal is detected, the sensor module undergoes initial processing locally at local level. Following this, the processed signal along with essential metadata such as time stamps, sensor ID and type is packaged for transmission. These metadata aid in contextualizing the data when further analyses are performed. The data packet also includes alerts if the sensor detects conditions that require immediate attention. These data packets are then transmitted to the edge server using wireless communication protocol which is IR in our project designed to minimize power consumption [14].

**Figure 4:**
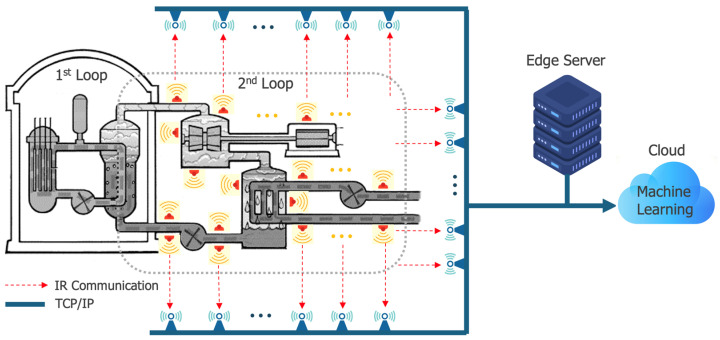
Sensors, receivers, edge server and cloud organization in the NPP monitoring system.

Figure 4 depicts the overall connection architecture of the edge computing-based remote diagnostic technology that we aim to implement in the secondary system of a nuclear power plant, as part of our project. Sensors are strategically placed at required locations within the secondary system and implement IR communication with receivers to collect the measured values. The receiver nodes that acquire data are connected via high-speed TCP/IP wired connections, facilitating the transmission of sensor data to the edge server. Simultaneously, the data are also forwarded to the cloud, enabling their utilization as machine learning data. 

The edge server acts as a midpoint between the sensors and the cloud. It performs more complex data-processing tasks than the initial processing done by the sensors. This includes data aggregation from multiple sensors, advanced analytics and machine learning models to interpret the data depending on the situation. Based on the processed data, the edge server makes immediate decisions. For instance, if a potential fault or anomaly is detected, the edge server can trigger immediate actions or controls directly to the operational technology, such as shutting down machinery or adjusting controls without waiting for cloud processing. Before sending data to the cloud, the edge server filters out unnecessary data and compresses them to optimize bandwidth and storage in the cloud. The edge server also ensures secure transmission of data to the cloud, which is planned to be equipped in the next stage of our project by deploying necessary encryption and security protocols to protect the data integrity and privacy. As for our development, we further discuss it in Section 5. 

The cloud plays a complementary role to the edge server. The cloud stores large volumes of data collected over time, which is impractical for local storage at edge servers due to space limitations. While edge servers handle real-time processing and immediate responses, the cloud provides resources for more resource-intensive analytics and machine learning models that require more computational power or historical data. The cloud enables centralized management of the edge server and sensors, allowing for updates, monitoring and coordination of policies across possible locations.

Together, edge sensors, the edge server and the cloud create a robust infrastructure capable of handling vast amounts of data efficiently, ensuring timely responses to continuous operational conditions and facilitating advanced analytics to drive decision-making [15]. This architecture ensures robust data communication and utilizes advanced computational resources to enhance monitoring and diagnostics in critical operations such as NPP.

## 4. Hardware Components of the Edge Sensor Module

### 4.1. Acoustic Sensor

Existing research on pipe leak-detection techniques based on deep learning technology has assumed a scenario where power is continuously supplied, allowing for the transmission of raw acoustic sensor data [16,17]. However, this study aims to minimize the amount of data transmitted from low-power wireless acoustic sensor modules to reduce power consumption. Previous studies have primarily utilized acoustic signals within the audible frequency range (below 20 kHz) to detect pipe leaks [18]. However, in power plants and industrial plants, considerable noise is generated within this audible frequency range, making it challenging to discern leak (anomalous) signals using this method. Therefore, this research applies a technique for detecting pipe leaks using signals in the 20 kHz to 100 kHz range, in accordance with international standard specifications (Class II) [19]. 

For low-power wireless sensing, it is crucial to address the issue of reduced acoustic signal-sensing capabilities arising from the use of low-power acoustic sensors and the power consumption associated with wireless communication. To mitigate these issues, we have enhanced signal-detection capabilities through the use of signal amplification. Furthermore, by converting the signal into the spectrum of a Fourier transform, we have successfully represented the characteristics of the signal with a minimal amount of data, thereby drastically reducing the volume of data required for wireless communication.

**Figure 5:**
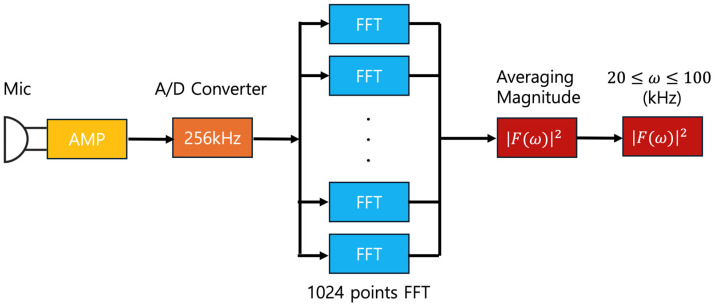
Proposed low-power acoustic sensor module.

The low-power sensor module developed for acoustic signal collection is described in Figure 5. The analog acoustic signals collected from the MEMS microphone (low power mode supply current: 75 µA, sensitivity: −38 dBV/Pa @ 94 dB SPL 1 kHz, frequency response: 100 Hz~80 kHz) are amplified and then converted into digital signals through an analog-to-digital conversion circuit (with a 100 kHz microphone, the sampling rate is 256 kHz). The sampled digital signals are transformed into frequency domain data through a one-dimensional discrete Fourier transform.

The one-dimensional discrete Fourier transform for the discrete function *f*(*x*), where *x* = 0, 1, 2, …, *N* − 1, is given by Equation (3),

(3) $$f\left(x\right)=\overset{N-1}{\underset{\omega =0}{\sum }}F\left(\omega \right)e^{j2\pi \omega x/N},\;x=0,\;1,\;\ldots ,\;N-1,$$

where $F\left(\omega \right)=\sum_{x=0}^{N-1}\frac{f\left(x\right)}{N}e^{-j2\pi \omega x/N},\;\omega =0,\;1,\;\ldots ,\;N-1$ and $e^{jx}=\cos x+j\sin x$.

The digital signals are collected at a high sampling rate of 256 kHz, which means 256,000 samples are taken per second. These collected signals are then divided into smaller segments. Each segment represents a time duration of 1/250 of a second. For each segment, we use 1024 of these samples to perform a one-dimensional discrete Fourier transform. Following the application of Equation (2), the magnitude of each Fourier transform is calculated, and the average values are obtained across 16 segments. The absolute values, representing the magnitudes of the frequency functions, are the spectra from the Fourier transform. This method allows for obtaining average spectral values across the 0–128 kHz frequency range at intervals of 0.25 kHz (equal to 256 kHz divided by 1024). According to the ASTM E 1002-05 standard [20], only the average spectrum for the frequency range of 20 kHz to 100 kHz is output, excluding information from both the audible and higher frequency bands. Consequently, the final data obtained through the low-power acoustic sensor module, as depicted in Figure 5, consist of average spectral values of the Fourier transform across the 20 kHz to 100 kHz frequency range at intervals of 0.25 kHz, totaling 320 data points. To reduce the data volume, the average spectral values from the Fourier transform are converted into 4 bytes float type per frequency. Consequently, the amount of data transmitted in a single session from the leak-detection sensor module, including an 8-byte header, amounts to (320 + 8) × 4 = 1312 bytes. If data were transmitted without refinement at a sampling rate of 256 kHz, the volume of data per transmission would amount to 256,000 × 4 = 1,024,000 bytes, or 1000 kilobytes. Therefore, the method proposed in this study reduces the volume of data transmitted by a factor of 1/800 compared to transmitting unrefined data.

### 4.2. Numerically Controlled Oscillator (NCO)

For the development of low-power acoustic sensing modules, it is essential to operate the frequency at which critical components, such as signal amplifiers and signal converters, function within a lower frequency band. This approach is necessitated by the fact that lower operating frequencies result in reduced current consumption. By bringing the operational frequency of these components to a lower band, it is possible to significantly decrease power usage, thereby enhancing the efficiency and sustainability of the acoustic sensing module. This strategy is crucial in extending the battery life and operational duration of wireless sensing devices, making them more practical for being used as edge sensors. In this case, the necessary mixing frequency signal is generated using the NCO, which produces a square wave rather than a sine wave in our project for simplicity and efficiency. For a square wave signal with a 50% duty cycle, Fourier analysis reveals the presence of odd harmonics such as the 3rd, 5th, 7th, etc. Due to these harmonics of the NCO, the mixing process also outputs signals that coincide with similar frequency vibrations. This overlap makes it challenging to isolate and observe signals within specific frequency bands. To address this issue, we filter the signals above the third harmonic out prior to the mixing process in the original signal. Also, the MCU operates at low power when set to a lower clock frequency and operating voltage. Considering the stable operation of the NCO and the data-processing capabilities, the clock frequency is set to 1 MHz, and it operates at approximately 60 µA from a 1.8 V power supply. This configuration optimizes the energy efficiency of the system while ensuring adequate performance for the required tasks. NCO operates by adding a fixed integer to its value at every clock cycle and altering the output signal when overflow occurs. By changing this integer, the frequency of the output signal can be adjusted. The CPU is incorporated with a 20-bit accumulator, which determines the output frequency based on the state of the 20-bit accumulator as in Equation (4):

(4) $$F_{nco}=\frac{F_{osc}\times V_{add}}{2^{21}}$$

In this context, *F_osc_* corresponds to the system clock frequency of 1 MHz. The use of 2^21^ instead of 2^20^ is because the state of the NCO needs to undergo two changes to complete one cycle. The addition value (*V_add_*; fixed integer), being an integer, introduces a maximum jitter of 1/*F_osc_*, resulting in a deviation from the 50% duty cycle of the generated signal, which consequently produces even harmonics. For the purpose of low power consumption in this system, a 1 MHz system clock was utilized to generate a 5 kHz signal with a maximum duty cycle error of 1%. The magnitude of the 2nd harmonics is approximately 0.01 relative to the magnitude of the generated signal, which is about the noise level when AI is learning or making judgments. The BPF range is 30–80 kHz. The 2nd harmonics fall within this range between 30 and 40 kHz. Even if mixing occurs, the signal itself is very small, and multiplying the small signal by 0.01 for the 2nd harmonics makes it negligible compared to the frequency components we aim to analyze. Even when the 2nd harmonics fall within the BPF range, they are generally smaller than the noise levels found in datasets typically used by AI. Additionally, the 2nd harmonics frequency can be further analyzed using the fundamental frequency.

### 4.3. Amplifier, Bandpass Filter, Mixer and Lowpass Filter

Amplifiers, Bandpass Filters (BPFs), Mixers and Lowpass Filters (LPFs) are fundamental components that work together to manipulate and refine signals to meet our technical requirements.

***Amplifier*** increases the power of the collected signals, boosting its voltage without altering its original frequency or waveform. This unit amplifies the output signal to detect relative changes, as it is difficult to distinguish between normal and anomalous signals based on the absolute magnitude of the sensor output, which is very small. This enhancement is critical to ensure that the signal can be processed effectively before it is fed to the mixer or ADC in the next stage. 

***Bandpass filter*** is responsible for selecting frequencies within the range of our target to pass through while blocking frequencies outside that range. It is used to isolate frequency components of interest from a broader spectrum of frequencies. The microphone’s response range is from 100 Hz to 80 kHz, and we have removed the audible frequency band (100 Hz to 20 kHz) to focus on the 20 kHz to 80 kHz range. The center frequency of this range is (20 + 80)/2 = 50 kHz. We utilized a band-pass filter (BPF) with a center frequency of 50 kHz and a bandwidth of 50 kHz. This filter features a ripple of 0.2 dB and is designed based on a 6th-order Chebyshev filter topology. The Chebyshev filter is chosen for its ability to provide a sharper roll-off and more selective frequency response, making it particularly suitable for applications requiring precise frequency discrimination. This configuration ensures that the filter effectively isolates the desired signal components around the center frequency while minimizing the influence of out-of-band noise. 

***Mixer*** combines two input frequencies to produce signals with output frequencies that are the sum *f*_1_ + *f*_2_ and difference *f*_1_ − *f*_2_ of the original input frequencies. This operation is known as frequency mixing [21]. Frequency mixing enables shifting signals from one frequency band to another to facilitate further processing or transmission. 

***Lowpass filter*** allows signals below a certain frequency to pass while attenuating signals above that frequency. We employed a low-pass filter (LPF) with a cutoff frequency of 125 Hz, which corresponds to half of our sampling frequency of 250 Hz. This filter features a ripple of 0.2 dB and is based on a 6th-order Chebyshev design. The choice of a Chebyshev filter allows providing better attenuation of frequencies beyond the cutoff point. This configuration effectively attenuates higher frequency components while preserving the integrity of the signal within the desired frequency range, thereby enhancing the signal-to-noise ratio and improving overall signal clarity.

**Figure 6:**
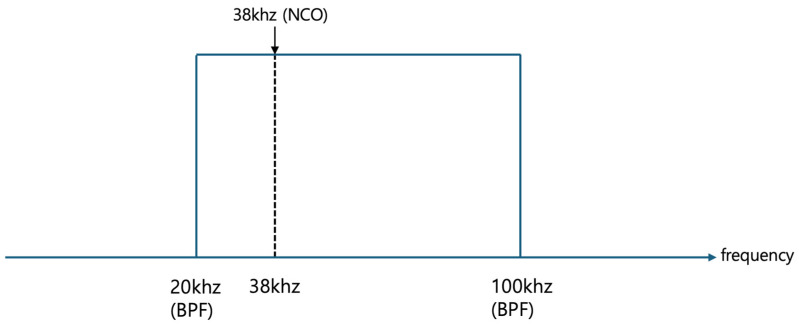
Constituting the 1st frequency band (20 kHz–100 kHz) using the bandpass filter.

Figure 6 illustrates the signal that has passed through a bandpass filter, depicting a specific frequency band. For example, this predefined frequency band could range from 20 kHz to 100 kHz, which constitutes the first frequency band. In instances where the target frequency for monitoring is 38 kHz, a numerically controlled oscillator can be configured to generate a signal at this specific frequency of 38 kHz.

**Figure 7:**
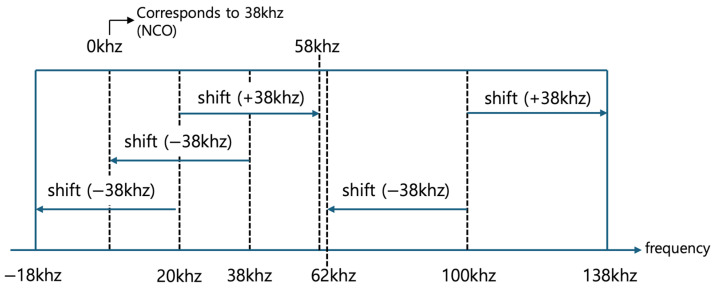
Frequency transformation into 2nd frequency band (−18 kHz–138 kHz) after mixing.

Figure 7 depicts the transformation of signals using the mixer to convert the predefined frequency band between 20 kHz and 100 kHz, into the second frequency band by mixing it with the target frequency of 38 kHz generated by the NCO. The mixer can convert two input frequencies into a signal with a frequency that is the sum of the input values. For instance, a 20 kHz signal that has passed through the bandpass filter is added to the 38 kHz frequency from the NCO, resulting in the shifted frequency of 58 kHz. Consequently, the signal, originally spanning 20 kHz to 100 kHz before mixing, is transformed into the frequency range of 58 kHz to 138 kHz after mixing.

Additionally, the mixer can also convert the input frequencies into a signal with a frequency that corresponds to the difference between them. For example, a 20 kHz signal can be reduced by 38 kHz to result in −18 kHz (this negative value indicates the shift below zero frequency, which may be theoretically considered but not practically useful), and a 100 kHz signal reduced by 38 kHz shifts to 62 kHz. Thus, the signals originally between 20 kHz and 100 kHz can also transform to span from −18 kHz to 62 kHz after mixing. Therefore, the signals processed by the mixer may overlap in the frequency bands from −18 kHz to 62 kHz and 58 kHz to 138 kHz. Moreover, any signal within the original 20 kHz to 100 kHz band at the specific frequency of 38 kHz, when reduced by the 38 kHz of the NCO, transforms to 0 Hz, indicating the baseline or null frequency shift corresponding to the pre-mixing monitoring target frequency of 38 kHz.

**Figure 8:**
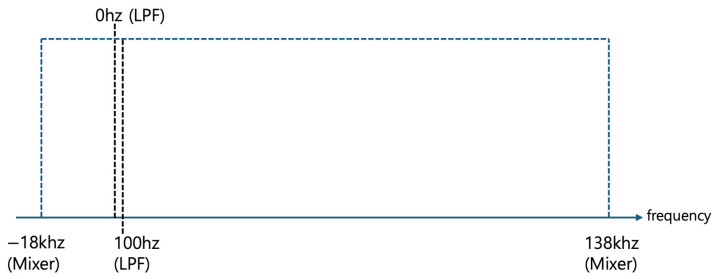
Isolation of 3rd frequency band (0 Hz to 100 Hz) using the lowpass filter.

Figure 8 illustrates the use of the lowpass filter to extract signals from the frequency range of −18 kHz to 138 kHz following mixing, isolating the third frequency band defined by the threshold frequency of 100 Hz or lower. Given that the threshold frequency is set at 100 Hz, the extracted third frequency band encompasses signals ranging from 0 Hz to 100 Hz. 

**Figure 9:**
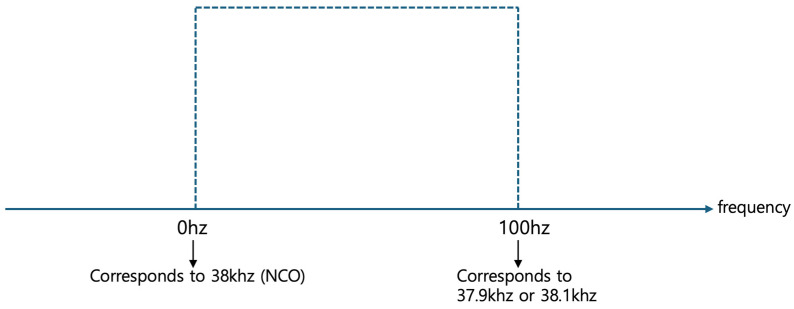
Transformed frequency band view around the target frequency of 38 kHz.

Figure 9 displays the expanded view of the frequency band from 0 Hz to 100 Hz after passing through the lowpass filter. As previously mentioned, 0 Hz corresponds to the pre-mixing monitoring target frequency of 38 kHz. Additionally, 100 Hz can correspond to a pre-mixing frequency of 38.1 kHz. Moreover, according to Equation (1), since cos(−100 Hz)= cos(100 Hz), 100 Hz can also correspond to a pre-mixing frequency of 37.9 kHz. Therefore, processing and analyzing signals within the 0 Hz to 100 Hz frequency band post-mixing is equivalent to handling signals within the 37.9 kHz to 38.1 kHz frequency band pre-mixing.

Consequently, by utilizing amplifiers and filters designed for handling the 100 Hz frequency band instead of those required for the 38 kHz frequency band, it is possible to reduce the power consumption of the wireless monitoring device significantly. 

### 4.4. Microcontroller Unit (MCU), Transmitter and ADC 

***MCU*** serves as the central processing unit within the system. In our project, we use 8-bit PIC series with MPLAB X IDE 6.20 platform, consuming 37 μA operating at 1 MHz. After the signal has been conditioned by the previous stage components, the MCU performs several key functions: It executes algorithms to further analyze or modify the signal by digital filtering, error checking and complex data transformations later in the edge server. Based on the processed data, the MCU makes decisions or calculations, such as detecting anomalies or triggering specific actions. In determining the presence of anomalous signals, we set the evaluation criterion to involve analyzing the spectral data between 30 kHz and 45 kHz. The methodology entails summing the spectral data values within this range and computing their average. Given that the data are sampled at intervals of 250 Hz, this results in a total of 320 samples. The threshold for anomaly detection is established based on the average value of the background noise under experimental conditions. For example, a signal can be programmed to be classified immediately as anomalous if its average deviates by 20% or 30% from the background noise average. Otherwise, the signal is considered normal. This criterion allows for a systematic and quantitative assessment of signal integrity, aiding in the accurate identification of potential issues. It also manages the operation of other components within the system, such as adjusting the frequency of the NCO or the operational parameters of the ADC and wireless transmitter. 

***Transmitter*** outputs the data to the server. Once the signal is processed and prepared actions are determined, the wireless transmitter’s role is to communicate the data or decisions to other devices or systems. This includes sending processed data, alerts or control messages over wireless protocols to the edge server, and the cloud server. 

***Analog to Digital Converter (ADC)*** is crucial for converting the conditioned analog signal into the digital format so that the MCU can process. It converts the analog signal received from the LPF into a digital signal by sampling the signal at 250 Hz rate and quantizing the amplitude into digital values. 

This orchestrated operation of amplifiers, filters, A/D converter, NCO, MCU and transmitters is what enables remote, reliable and safe monitoring and control of our target NPP system.

## 5. Implementation and Test Results 

**Figure 10:**
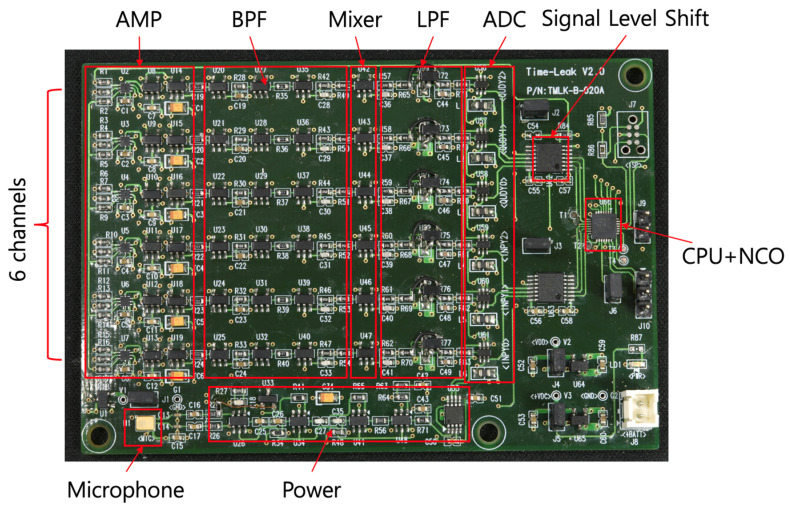
Implementation of the 6 channel acoustic signal-acquisition sensor module.

For the edge sensor module described above, designed for monitoring NPPs, it is possible to collect acoustic signals from a single frequency band of interest at low power. According to our technical report, anomalous signals in nuclear plants are measured typically around the high-frequency region of 38 kHz [22] since it is difficult to distinguish from noise in the audible frequency range. Consequently, we have assumed that anomalies may arise within six frequency bands near 38 kHz, thus have constructed a test board capable of simultaneously extracting acoustics from these six frequency bands. Figure 10 illustrates the implementation of our target test board designed for the simultaneous real-time acquisition of acoustic signals across six frequency bands from 6 channel sensor modules. The components enclosed in red boxes are identical and arranged vertically in six columns, demonstrating the systematic layout of AMP-BPF-Mixer-LPF-ADC in six separate lines. In the lower left part of the figure, a shared microphone serves as the input for all six channels, simultaneously delivering signals to each channel which then detects signals within specific frequency bands. On the right, the CPU and NCO continuously manage the six channels to convert their frequencies into low-frequency, low-power signals. Ultimately, the data extracted from these six channels are prepared for transmission to the next stage of processing involving edge servers and the cloud.

**Figure 11:**
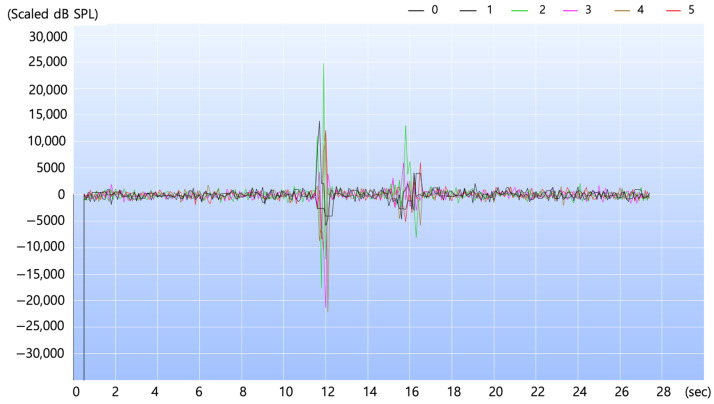
Six anomalous acoustic signals collected from six frequency bands near 38 kHz.

Figure 11 displays six acoustic signals collected before communication from six frequency bands near 38 kHz, each represented in a different color. These signals are considered normal until they meet the criteria for determining an anomalous state as described above. However, if these signals exceed the criteria for determining the anomalous state, the signals in that specific frequencies are initially classified as anomalous signals and are then transmitted to the edge server. Subsequently, more complex artificial intelligence algorithms are applied at the edge server to assess the certainty of the anomalous signals. In determining the occurrence of leaks during deep learning analysis, we use the Shapley additive explanations (SHAP) technique [23]. When a leak occurs, it is observable as a pronounced signal as in the center of the figure. Such changes in signal magnitude enable the detection of potential anomalies. The set of suspected signals is then transmitted to a remote diagnostic edge server, where artificial intelligence classification is employed to assess the authenticity of the leaks. The key frequencies include 37.6 kHz, 37.8 kHz, 38.0 kHz, 38.2 kHz, 38.4 kHz and 38.6 kHz, showing the intensity of the signals over time. During the experiment, artificial high-frequency noise was introduced around 12 s and 16 s to generate anomalous signals. The intensity of the signals is measured in dB SPL [24], which is the output from the acoustic sensors. However, the original sensor output was too low in magnitude for effective analysis, prompting us to scale up the signal intensity values to enhance visibility. It is important to note that the specific values on the y-axis are not as significant as the relative changes in signal intensity, which carry meaningful information.

The measured current is approximately 2–3 mA, which translates to about 333 µA to 500 µA per channel. This is slightly below the initially anticipated range of 200 µA to 300 µA per channel Based on the current measurement results, using a 9000 mAh battery results in an operating time of 3000–4500 h which is slightly more than 4–6 months. We expect to extend the operating time by such methods as switching from the PIC MCU to an ARM M0 processor and reducing the power consumption in the mixer section, which draws more current than initially anticipated. If the original high-frequency signal were to be used without refinement, employing high-frequency amplifiers and filters, it would consume over 80 mA of current.

**Figure 12:**
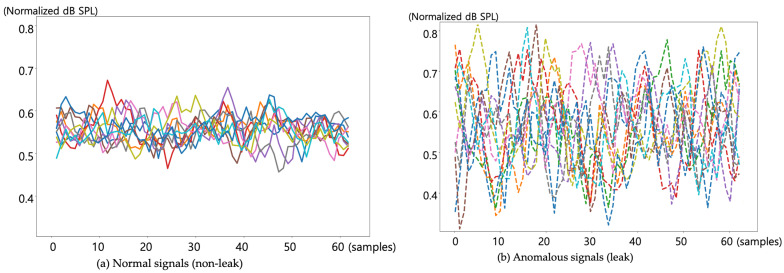
Ten groups of 64 samples of normal and anomalous signals for deep learning training.

Figure 12 illustrates the process of applying sensor data to deep learning by using a single channel. The dataset comprises 24,234 normal signal samples and 24,194 abnormal signal samples. These samples were segmented into sequences of 64, resulting in the total of 378 normal samples and 378 abnormal samples respectively, for deep learning training and testing. This dataset is then divided into training and testing sets in an 8:2 ratio, yielding 614 samples for training and 152 samples for testing. We employed a deep learning setup for our experiments, which consisted of training the model over 100 epochs with a batch size of 32. The learning rate was set at 0.005, and we utilized the Adam optimizer for optimizing the network parameters. The architecture of our Feedforward Neural Network (FNN) included an input layer with 64 neurons, followed by three fully connected (FC) layers. The first FC layer had 64 input neurons and 50 output neurons, followed by a ReLU activation function and batch normalization. The second FC layer consisted of 50 input neurons and 20 output neurons, also followed by a ReLU activation function and batch normalization. The third FC layer contained 20 input neurons and 5 output neurons, again followed by a ReLU activation function and batch normalization. The final output layer was a linear layer with 5 input neurons and 2 output neurons, followed by a softmax activation function to produce the final class probabilities. We conducted our experiments using TensorFlow 2.3.1. Notably, our evaluation process did not include a validation step; instead, we assessed the performance of our deep learning models solely using test datasets. The figure displays the signal intensities of 10 segments of (a) normal signals and 10 segments of (b) anomalous signals plotted against the sample count (time axis). Additionally, since the exact values of signal intensity are not crucial, normalization was applied to demonstrate the relative sizes of the signals. The x-axis represents these batches of 64 samples each. It can be observed that the data in the anomalous state are generally larger in magnitude and exhibit more significant fluctuations compared to the data in the normal state.

**Figure 13:**
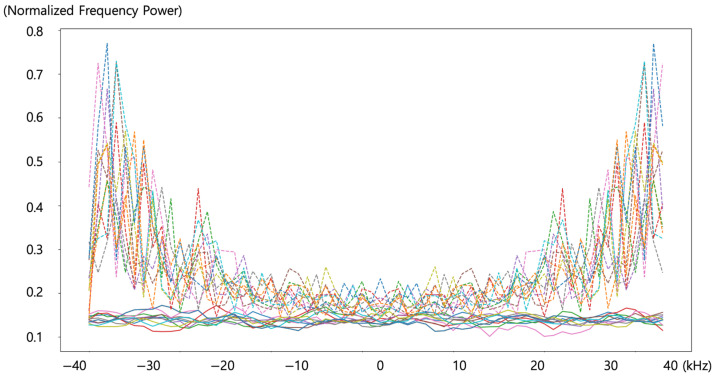
Power spectra of 10 groups of 64 samples of normal and anomalous signals.

To better illustrate these characteristics, Figure 13 applies Fourier transform to display the data as power spectra. For the normal state data, the Fourier transform shows a relatively uniform magnitude across the entire frequency spectrum. In contrast, the data in the anomalous state show greater magnitudes in the higher frequency ranges, with a particularly noticeable peak around 38 kHz.

An artificial intelligence model for determining the outbreak of leaks, based on signals collected via the time-domain-based wireless sensing module, was designed using a Fully connected Neural Network (FNN). This model incorporated two additional reinforcement factors: Gaussian random Fourier features and Fourier transformation. A comparison of the time-domain signals depicted in Figure 12 with their Fourier-transformed counterparts in Figure 13 reveals that distinctions between leak and normal states are more clearly discernible in Figure 13. By employing Fourier-transformed data during the preprocessing stage to assess the presence of leaks, the model achieved an accuracy of 100% as shown in Table 1.

## 6. Conclusions

The development and implementation of the novel low-power wireless monitoring system designed specifically for nuclear power plants as presented in this paper signifies a substantial advancement in the field of nuclear facility management and safety. By integrating state-of-the-art signal-processing techniques and leveraging energy-efficient technologies, this system not only addresses the immediate operational challenges posed by the hostile environments of nuclear power plants but also sets a new standard in proactive risk management and operational efficiency. Throughout this study, we have developed a low-power sensor system based on the heterodyne method, utilizing an MCU and an NCO, capable of selectively examining signals across six frequency bands. Unlike traditional systems that process signals from all six frequency bands simultaneously, our proposed system employs a round-robin approach. This method sequentially transmits signals from each of the six frequency bands, thereby significantly reducing power consumption. Additionally, our system offers flexibility in adapting to changes in the frequency band of interest. When the sensor module detects an anomalous signal, it transmits this data to an edge server, where AI models reliably assess and confirm the presence of the anomaly.

Moreover, the edge sensor operations, utilizing the combination of our target-specific hardware components and sophisticated data-processing algorithms, demonstrate our robust capability to not only detect and analyze signals but also to transmit critical data securely and efficiently to both edge servers and cloud-based systems. We plan to address the hierarchical data processing in our subsequent research. Our future work will involve comprehensive experiments to demonstrate the transmission of high-bandwidth data in industrial environments characterized by electromagnetic pollution. This challenging task will be a key focus, and we aim to provide robust experimental evidence to support our claims regarding the reliability and efficiency of our data-transmission methodology under such conditions. This will ensure that decision-making is both timely and informed, thereby significantly reducing response times to potential faults and hazards. The practical tests and deployment within a controlled environment have shown that the proposed system can significantly reduce the dependency on traditional wired networks and frequent human intervention, lowering both the risk to human life and the overall cost of maintenance. The extended battery life and reduced power consumption also align with the broader objectives of sustainability and environmental responsibility in energy production.

In conclusion, the adoption of this low-power wireless monitoring system in nuclear power plants not only enhances operational safety and efficiency but also supports a transition towards more autonomous and resilient nuclear facility operations. Future work will focus on scaling the implementation of this technology to encompass a broader range of sensors, adopting lower-power MCUs and power saving modes and integrating more advanced machine learning models to further enhance the predictive capabilities and the consuming power of the system. Also, we plan to check the current of each component in the mixing stage separately to identify the cause of leaking current, minimize the input signal to the mixing stage, replace capacitors with those that have lower leakage and improve the circuit layout and PCB materials to suppress leakage currents as much as possible. This ongoing evolution of monitoring technologies is expected to play a crucial role in the future of nuclear energy management, ensuring that safety and efficiency are maintained at the highest standards.

## Figures and Tables

**Table 1:** Comparative performance of AI models for leak detection.

Deep Learning Model	Performance (%)
Fully connected Neural Network (FNN)	97
Gaussian Random Fourier Feature + FNN	98
Fourier Transformation + FNN	100

## Data Availability

The datasets generated from the current study are available from the corresponding author upon reasonable request.
